# Use of a non-invasive accelerometric method for diagnosing gastroesophageal reflux in premature infants

**DOI:** 10.1038/s41372-021-01034-5

**Published:** 2021-03-23

**Authors:** Ira H. Gewolb, Frank L. Vice

**Affiliations:** Division of Neonatology, Department of Pediatrics and Human Development, Michigan State University College of Human Medicine, East Lansing, MI USA

**Keywords:** Translational research, Gastro-oesophageal reflux disease

## Abstract

**Objective:**

To evaluate the clinical usefulness of a non-invasive accelerometric device to diagnose GER in preterm babies.

**Study design:**

An accelerometer was taped over the sub-xiphoid process in 110 preterm (GA 29.6 ± 3.3 wk) infants (133 studies). Low frequency, sub-audible signals were captured via digital recording (sampling rate 200 Hz), then re-sampled (rate = 60 Hz) to create a spectrogram (focused range 0–30 Hz). Mean amplitude in the focused range was calculated.

**Results:**

Of 85 studies with simultaneous pH-metry and accelerometry, 18 had concurrent positive and 23 had concurrent negative scores, 42 had negative pH scores when accelerometry was positive (≥1 µV), consistent with non-acid reflux. Eleven infants at high risk of aspiration received surgical interventions. All but 1 had negative pH scores while 10/11 had positive accelerometry.

**Conclusions:**

The non-invasiveness of this accelerometric technique allows for GER screening and for repeated testing to assess efficacy of interventions.

## Introduction

Gastroesophageal reflux (GER) is thought to be the result of retrograde passage of liquified or aero-gastric contents into the esophagus and above, generally believed to be precipitated by the transient relaxation of the lower esophageal sphincter [1, 2]. GER has especial importance in preterm infants and other populations unable to communicate their symptoms and who are at risk for aspiration or micro-aspiration of liquid contents [3, 4]. Similar concerns are present in other at-risk populations, such as children and adults with obesity, respiratory or neurological disease, as well as the geriatric population [5–7]. The symptoms of GER are different in preterm babies compared with adults and older children and are far more likely to involve non-acid reflux [8–10]. The risk of aspiration is the main concern in preterm infants whereas erosion of the esophageal lining and a potential increase in cancer risk are long-term issues in adults.

Diagnostic modalities in GER are generally characterized by invasiveness and often a lack of reproducibility [11–15]. In preterm babies the pH probe, until recently, was the most commonly used diagnostic method even though its use has decreased in the past few years, to be replaced by the multiple impedance monitor/pH probe (MII-pH). GER is most commonly non-acid in nature [8–10], yet, by definition, pH probes used alone do not diagnose non-acid reflux. pH probes, multiple impedance monitors, endoscopy (in adults), and imaging techniques (such as upper GI studies, swallow studies, etc.) sometimes used in evaluating GER are invasive, involving placement of an esophageal probe and often the use of X-rays to confirm proper placement, making repetitive use problematic. Swallow studies and ultrasound only capture brief periods of time and may overdiagnose or miss positive episodes of GER, and are not recommended for the diagnosis of GER. The “wireless” system for esophageal pH monitoring [16] involves placing an antimony electrode attached surgically to the mucosal wall of the esophagus, which then transmits a signal via a pH telemetry capsule to an external receiver. Thus, it is also invasive and is based on pH, with the inherent problems with pH probes [11, 12, 14,17–20].

Esophageal pH is not reliable in diagnosing GER in premature infants because of a higher baseline gastric pH due to milk feedings that neutralize acidity [17–19]. Multiple impedance monitoring also suffers from lack of reproducibility [14, 15] and, until recently, lack of standards for neonates. Indeed, even the most recent attempt to provide reference standards for infants [21] did not study preterm infants and did not study normal healthy children because of ethical concerns over the invasiveness of the tube placement. The invasiveness of most techniques makes it difficult to do repeated studies to assess and modify treatment options. Proof of concept for our accelerometric device has been recently published [22]; the current study represents the first attempt to use our GER diagnostic device in a clinical setting.

## Methods

**Fig. 1:**
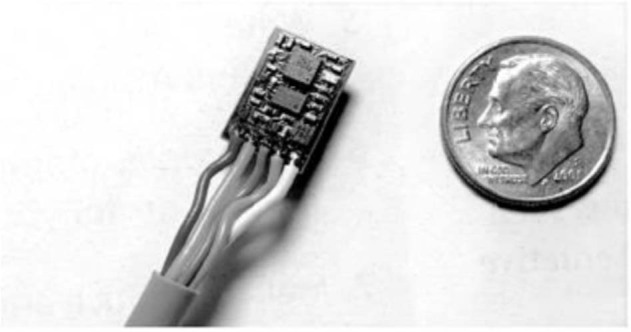
Accelerometer. The accelerometer currently used in these studies (see Methods for details). An American dime (diameter = 17.91 mm) is shown for size comparison purposes.

An accelerometer (Honeywell Sensotec MAQ36; Columbus, OH) (Fig. 1) was taped to the skin over the sub-xiphoid process. Using a 200 Hz sampling rate, signals were captured on a DASH 2EZ + digital recorder (Astro-Med, Inc. West Warwick, RI). To eliminate other NICU electronic equipment interference, a band stop filter at 60 Hz was used. More recently, we have used a custom-designed digital recorder in conjunction with a digital three-dimensional accelerometer (Freescale MMA8451Q, Mauser Electronics, Mansfield, TX) [22]. In most babies a concurrent 5-French single channel pH probe (pHNS-P, ComforTEC ™; ZepHyr 2000-A monitor, Sandhill Scientific, now Diversatek Healthcare, Milwaukee, WI) or Sleuth ZPN-BS-46 impedance monitor with a 6.4 French probe (ComforTEC™; Z07-2000B impedance/pH monitor, Sandhill Scientific, now Diversatek Healthcare, Milwaukee, WI) was in place.

The recorded signal was processed on Sigview 3.2. (SignalLab, Pforzhein, Germany) using a Fast Fourier Transform (FFT) with a segment size of 1024. The signal was then de-jittered by subtracting 200 µV (if the result was negative, it was counted as 0). To eliminate any potential artifact, if any remaining segment amplitude above 60 Hz exceeded 200 µV the entire segment (from 0–100 Hz) was excised. The data was then re-sampled at 60 samples per second to create a spectrogram with a segment size of 512 and a focused range of 0–30 Hz. The mean amplitude in µV was then calculated. An average accelerometric value of ≥1 µV for the entire recording period (3–6 h) was preliminarily chosen as “abnormal.” This was derived from a “best-fit” of the data, which would minimize occasions when a negative accelerometric score occurred when there was a positive Boix-Ochoa pH score (cut-off = 16.6) [23].

Pre-established inclusion criteria included all premature babies <37 wks GA. Exclusion criteria included all babies with upper gastrointestinal tract abnormalities, gastrointestinal obstruction, or prior gastrointestinal surgery. Most infants were enrolled because they were suspected of having GER and were scheduled for a pH probe or an MII-pH study. Approximately 25% of infants were not suspected of having GER and were enrolled without a concurrent pH or MII-pH study, in order to begin to establish values for “normal” preterm infants. Informed consent was obtained prior to each study. This protocol was approved by the IRBs of Michigan State University and Sparrow Hospital.

## Results

A total of 110 preterm infants (<37 wk GA) with 133 studies were enrolled. 85 had pH probes at the same time as the accelerometric recordings. The cohort had a mean gestational age of 29.6 ± 3.3 (SD) and a mean birth weight of 1458 ± 781 gms and were studied at 68.3 ± 39.8 days postnatal age (39.2 ± 4.0 wks post-menstrual age (PMA)). These were further divided into babies <30 wks GA (*N* = 59; 66 studies) and those between 30 and 36^6/7^ weeks (*N* = 51; 67 studies). There was no statistical difference in the PMA at which the studies were done (*p* > 0.05), so for the purposes of this study the data were combined. 64.4% were male and 82% were Caucasian. 21% were on caffeine at time of study (Table 1).

**Table 1:** Patient characteristics.

	<30 weeks	30-36 6/7 weeks	Total population
*N* = studies/infants	66/59	67/51	133/110
Birth weight (gm)	985 ± 287	1940 ± 808	1458 ± 781
GA (wks)	26.8 ± 1.8	32.2 ± 1.8	29.6 ± 3.3
Postnatal age @ time of study (d)	80.1 ± 33.8	48.8 ± 35.8	68.3 ± 39.8
PMA @ time of study (d)	39.4 ± 3.5	39.0 ± 4.4	39.2 ± 4.0
GER meds* prior to study (%)	25.5	21.1	23.5
Caffeine prior to study (%)	21.0	21.0	21.0
Gender (%)	M = 64.4	F = 35.6	
Race: Caucasian	82.8%		
African-American	8.6%		
Hispanic	6.3%		
Asian	1.6%		
Other	0.8%		

*Reglan (metochlopramide), zantac (ranitidine), or prevacid (lansoprazole).

**Fig. 2:**
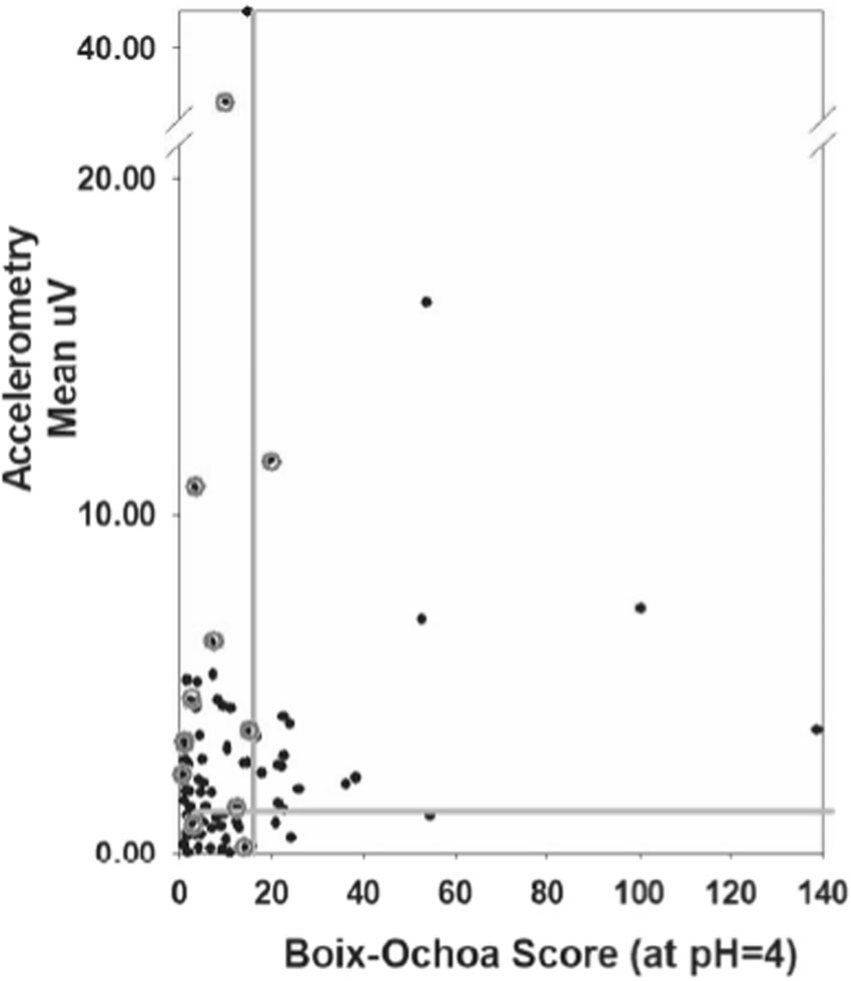
Concurrent accelerometric and pH recordings in preterm infants suspected of having GER. The gray lines indicate the cut-off between normal/abnormal (Boix-Ochoa = 16.6 and the accelerometric score = 1 µV). Data points in the upper right-hand corner are instances of concurrent positive studies using pH probes and accelerometry. The lower left-hand corner represents co-temporal negative studies. The upper left-hand studies are cases where the pH probe was negative when our method was positive; this likely represents cases of non-acid reflux. There were two points where our method was negative even though the pH probe was mildly positive. The circled points represent cases where the medical and surgical teams deemed that the baby was at high enough risk for aspiration to require surgical intervention (g-tube/fundoplication, tracheostomy). Note that while almost all these infants had positive accelerometric scores, most had negative pH scores.

The comparison of concurrent accelerometric (mean µV) and pH recordings (Boix-Ochoa scores [23]) is shown in Fig. 2. The gray lines indicate the cut-offs between “normal” and “abnormal.” There were 18 occasions when the Boix-Ochoa scores were “positive,” (>16.6, indicative of significant acidic GER) and the accelerometric recordings (>1 µV) were also indicative of pronounced GER. There were 23 studies that were negative using both methodologies. However, when the pH recordings had “negative” Boix-Ochoa-scores, many of the accelerometric recordings in the premature infants were positive (*n* = 42), consistent with the fact that the majority of the reflux episodes in preterm infants are non-acidic [17–19]. Overall, 42 of the 60 (70%) studies that were positive using our methodology had a negative Boix-Ochoa score, consistent with previous studies showing that ≈75% of GER is non-acidic in preterm infants [9, 10] including studies comparing MII with pH scores [8, 14, 21, 24, 25].

**Fig. 3:**
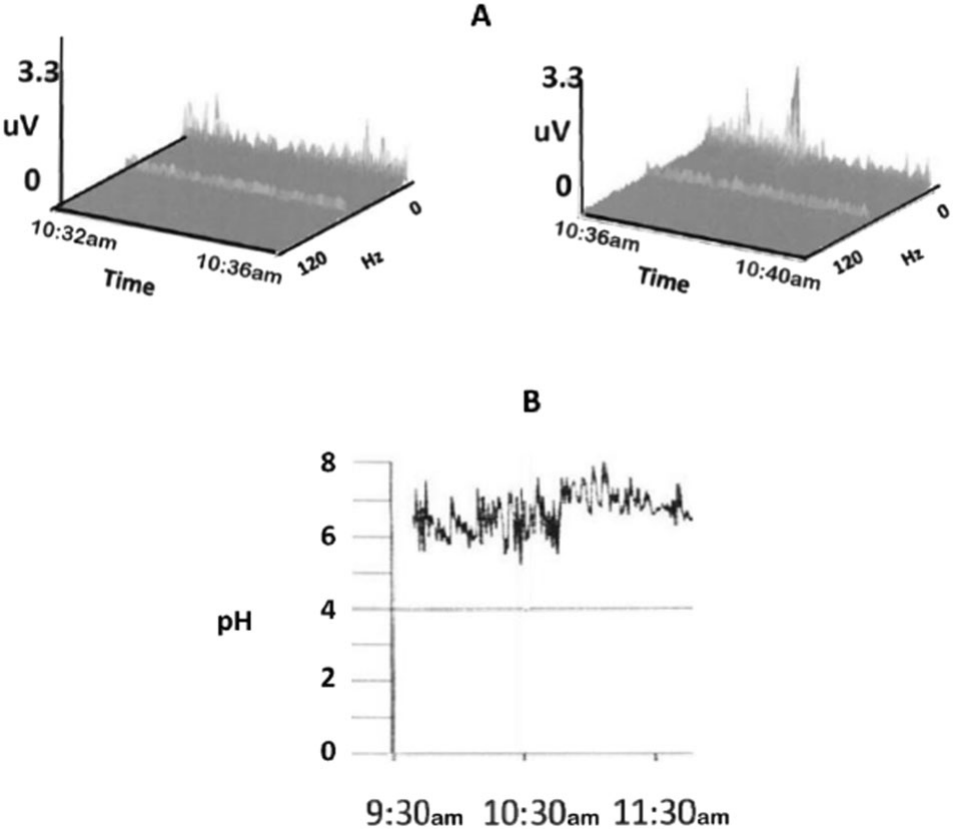
Positive accelerometry reading during non-acid reflux (no change in pH). Simultaneous pH probe (**B**) and accelerometric recording (**A**) were performed. Note that the pH remained between 6 and 8 for a 2-hour period between 9:30 and 11:30 a.m., at the same time that the accelerometric recording shows movement indicative of GER (during the time period between 10:32 and 10:40 a.m.).

An example of a positive accelerometric signal occurring during a period of non-acidity (high pH) is shown in Fig. 3. Figure 3A depicts a positive accelerometric recording, while Fig. 3B shows a concurrent pH probe reading of ~7.

There were two studies where our method was negative even though the pH probe was mildly positive, yielding a positive predictive value of 90% (18/20).

Of great importance, we noted that of the 11 children deemed by the surgical and medical teams to require surgery (G-tube, fundoplication, or tracheostomy) for risk of aspiration (depicted as gray circles in Fig. 2), 8 had “false-negative” Boix-Ochoa scores at the same time that positive accelerometric scores were obtained, suggesting greater predictive accuracy with the accelerometric methodology. In two of the surgical cases both methods were positive and in one case, both were negative.

There were 20 children who were treated with anti-GER medications (reglan, prevacid, or zantac) prior to the pH/accelerometric study (See Table 1). Of these, 19 of 20 had a negative pH score (<16.6). In seven cases, the accelerometric score was also negative, but in 13 accelerometry was positive, either indicating true non-acid reflux or a false negative pH score induced by the antacid therapy. In none of these 20 cases was there a positive Boix-Ochoa score paired with a negative accelerometric value.

The accelerometric signal could often be elevated for many minutes, as was also seen in our previously reported concurrent ultrasound recordings [22], which showed an actual back and forth movement of the refluxate in the lower esophagus. Accelerometry during feeding generally did not register as positive, suggesting that unlike retrograde reflux, normal peristaltic movements were not robust enough to register on our device.

**Fig. 4:**
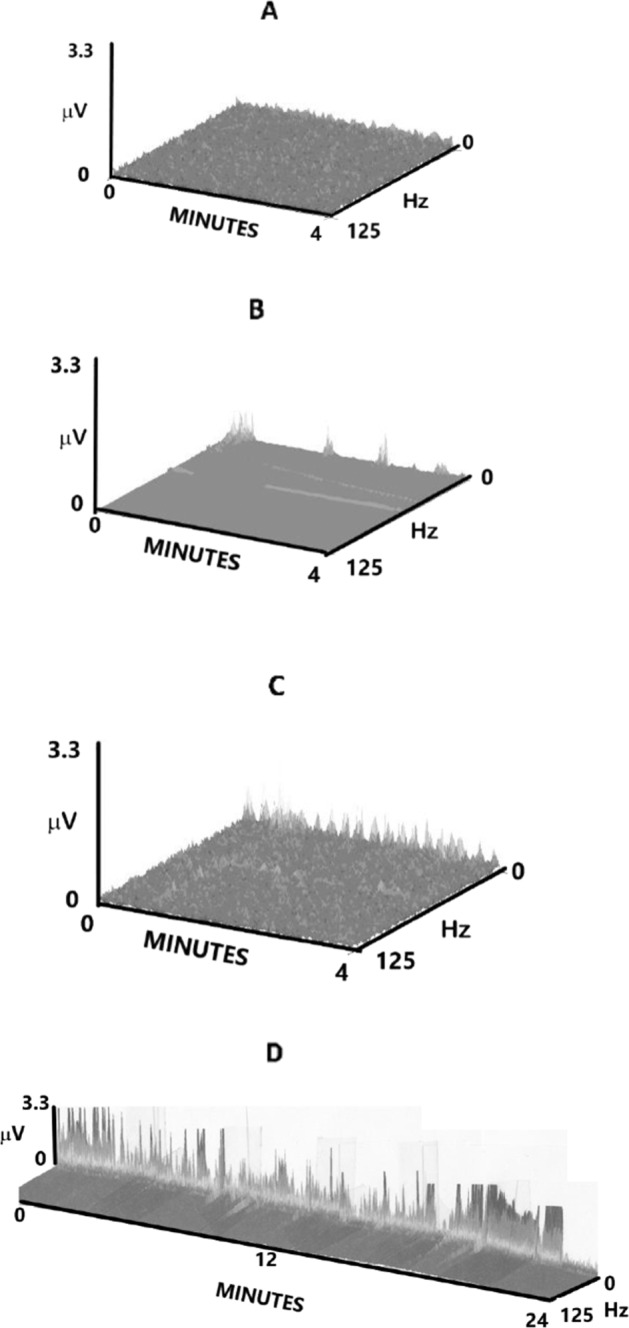
Accelerometric patterns. **A**. A negative 4-minute accelerometric sequence. **B**. An intermittently positive accelerometric recording. **C**. A rhythmic up-and-down accelerometric reading. **D**. A 24-minute recording with continuously positive accelerometric deflections. *x*-axis = 0–4 minutes (4**A**–**C**); 0–24 minutes (4**D**), *y*-axis = 0–3.3 µV, *z*-axis = 0–125 Hz.

Figure 4 depicts some different patterns obtained by our accelerometric method. Figure 4A demonstrates a negative 4-minute sequence, with baseline accelerometric readings throughout. Figure 4B shows intermittent positive accelerometric deflections during a 4-minute recording. In Fig. 4C, we see up-and-down waveform accelerometric deflections, possibly representing back-and forth movements in the esophagus, as we have previously demonstrated on ultrasound recordings [22]. These appear to occur in a rhythmic manner (≈4/minute) and may represent stomach muscle contractions, resulting in reflux passing through an open lower esophageal sphincter. Figure 4D shows accelerometric movements occurring continuously for >20 min. These movements suggest that the lower esophagus and sphincter are open for this duration of time.

## Discussion

Accelerometry measures the rate of change of velocity; an accelerometer is a sensor designed to generate an electrical signal in response to acceleration, parallel with its sensitive axis. Our uniaxial accelerometer measured acceleration in the *z*-axis (perpendicular to the chest wall). This suggests that we are assessing upward movement of the esophageal wall as it is filled by liquid refluxate (but possibly not by air reflux/burping). Between nutritive swallows (or reflux) the esophagus is collapsed, but the lumen can distend in the anterior–posterior axis and also laterally to accommodate a bolus [26].

We have previously demonstrated proof of concept of our GER diagnostic device when compared with pH probes, ultrasound, and impedance monitoring [22]. In the current study we show that our accelerometric monitor can non-invasively diagnose GER events in preterm infants suspected of having GER(D) (regardless of the acidity of the refluxate) with an accuracy that is comparable with, and may be superior to, the pH probe technique, and may be useful as a screening test prior to using the more invasive MII-pH recording. Our accelerometric GER monitor also allows for repeated longitudinal monitoring of babies suspected of having GER Disease (GERD), to see whether an intervention is working. The discomfort of inserting a naso-esophageal tube is also circumvented, which would also be especially important in older children and adults; and would prevent young children from constantly trying to pull the tube out.

Recordings could also have been obtained by placing the accelerometer on the back of the patient, closer to the esophagus, which is posterior to the trachea and the heart. However, we chose not to do so, as current AAP recommendations (for the prevention of Sudden Infant Death Syndrome) are for the babies to sleep supine [27].

We chose an accelerometric score of ≥1 µV to indicate significant GER. This was an arbitrary choice to best fit the data in Fig. 2. Future studies will allow us to see whether this score is valid or if it must be adjusted. We are in the process of developing a digital algorithm that will use other characteristics of the data (i.e., % time spent ≥1 µV, highest voltage, longest episode, number of episodes, etc.), to yield a score with potentially greater accuracy.

We found a 90% correlation with positive pH probe results (Fig. 2). Since we were constrained by the fact that at present our device can only collect a maximum of 6 h of continuous data, which is then compared with an ≈24 hr pH recording, it is certainly possible that the accelerometric score might not be reflective of a full 24 h of a pH probe recording. However, we could not simply match our shorter recordings with the concurrent hours of a pH or MII-pH probe, since pH recordings of <24 h are not considered valid by Diversatek (Milwaukee, WI) and certain elements of the pH scoring algorithm (number of episodes, longest episode, % time pH <4) will yield different scores at <24 h.

When the pH probe was negative our device could be negative or positive. Our monitor does not depend on the acidity of the refluxate. We believe our positive results in this situation are not errors but represent those premature infants in whom reflux is non-acid [8–10,14, 21, 24, 25]. This is supported by the fact that infants deemed clinically to require GER surgery (gastrostomy-tube or fundoplication, rarely tracheostomy) were almost always positive using our device, but were generally negative by pH probe (set at <4) (see Fig. 2, gray circles). Some of the pH probes might be read as positive based on a sawtooth pattern indicating a decline in pH (but not reaching pH = 4) spontaneously or after a feed, but this then requires a visual post-hoc qualitative reinterpretation of the pH results, since the commercial pH algorithm reports the results as negative. Potentially, the slope or rate of rise of a GER-associated episode may differentiate between different types of regurgitation events; this has not yet been tested.

The accelerometric signal could be positive intermittently or could last for many minutes (Fig. 4D), as was also seen in our previously reported concurrent ultrasound recordings [22]. At present it is not clear whether a pattern of longer duration represents a higher risk of aspiration to the infant, but future follow-up testing could clarify this important question.

Feeding did not seem to result in significant changes in accelerometric scores; we speculate that reflux movements are more robust than normal feeding peristalsis in premature infants, especially those receiving smaller volume intermittent feeds or those with continuous tube feeding directly into the stomach. Indeed, esophageal manometric studies in preterm infants between 30 and 34 weeks PMA have documented a lower esophageal peristaltic amplitude and velocity than term infants [28, 29].

GER in neonates has been associated with episodes of vomiting, poor feeding and growth delay, and aspiration with resultant respiratory symptoms [11]. (Presumably, the main risk is from liquid contents, as it is difficult to see how reflux of gas can cause aspiration or vomiting). Diagnosis based on clinical symptoms is not accurate, leading to a 13-fold variation in the diagnosis of GER in different NICU’s [30]. A large proportion of all NICU graduates are discharged on anti-GER medications, often for many months [31–33].

MII-pH monitoring, the current gold-standard, also requires a naso-esophageal tube and often confirmatory placement X-rays. Positive impedance scores are also poorly associated with symptoms of GER [14, 15]. Intraluminal impedance also misses some reflux episodes detected by pH probes [34]. It is true that the impedance methodology allows an assessment of the height of the refluxate [11], which might, by extension, reflect the risk of aspiration. While at present, we are only using one sensor on our device, we are in the process of developing an array of accelerometers, which will give us a similar ability of assessing the height of the refluxate. Most pharmacological treatments, besides having questionable efficacy and a variety of side-effects [35], have not been studied longitudinally. The non-invasiveness of the accelerometric methodology allows it to be used repeatedly without discomfort.

There are a number of limitations to the current accelerometric device that need to be addressed before it can become an adjunct to diagnosis. Recording time needs to be extended to a full 24-hour period, although in neonates who are fed 6–8 times a day, this may not be necessary. The validity of the 1 µV cut-off needs to be established, as do normal cut-offs at different ages, from preterms to older adults. An automated algorithm needs to be developed and validated, as does the proposed accelerometric array. Finally, the device needs to be tested in other populations at risk for GER-related issues.

## Conclusion

Accelerometry appears to be capable of diagnosing GER in preterm infants. A non- invasive screening tool could be important, especially in cases where etiology or response to therapy is not clear, as we believe that GER remains a significant risk for aspiration in preterm infants. Its use as a screening tool could also be helpful in adults with GER-like symptoms who do not respond to first line therapy. This device could also be used to screen other populations including the pediatric population, adults and children with neurological problems, and geriatric patients.
